# Phylogeny-informed random forests for human microbiome studies

**DOI:** 10.1128/spectrum.03451-25

**Published:** 2026-03-24

**Authors:** Hyunwook Koh

**Affiliations:** 1 Department of Applied Mathematics and Statistics, The State University of New York-Korea (SUNY Korea) 540304, Incheon, South Korea; University of Arkansas for Medical Sciences, Little Rock, Arkansas, USA

**Keywords:** random forest, phylogenetic clustering, localization, feature selection, feature weighting, human microbiome

## Abstract

Random Forest is a widely used tree-based ensemble learning algorithm that efficiently captures complex nonlinear relationships and higher-order feature interactions with no distributional assumptions to be satisfied. It is also well-suited to human microbiome studies, where the data are highly skewed, overdispersed, discrete, and irregular. Here, I pay particular attention to the phylogenetic tree information that reflects evolutionary ancestry and functional relatedness among microbial features. Proper incorporation of phylogenetic tree information into microbiome data analysis has provided new insights and improved analytical performance. In this paper, I introduce an extension of the Random Forest algorithm that incorporates phylogenetic tree information, named Phylogeny-Informed Random Forests (PIRF), to improve predictive accuracy in human microbiome studies. The core mechanism of PIRF lies in its localized approach; rather than treating all features as competing globally to be selected or weighted, PIRF identifies informative features within each phylogenetic cluster (i.e., a localized group of microbial features that are evolutionarily and functionally related), thereby enriching functional representations while reducing tree-to-tree correlation. I demonstrate the high predictive accuracy of PIRF, compared with other off-the-shelf tools, across seven benchmark tasks: four classification problems (gingival inflammation, immunotherapy response, type 1 diabetes, and obesity) and three regression problems (cytokine level, age based on oral microbiome, and age based on gut microbiome).

**IMPORTANCE:**

PIRF is an extension of the Random Forest algorithm that incorporates phylogenetic tree information to improve predictive accuracy in human microbiome studies. PIRF can serve as a useful tool for microbiome-based disease diagnostics and personalized medicine. The software and tutorials are freely available as an R package, named PIRF, at https://github.com/hk1785/PIRF.

## INTRODUCTION

Decision Tree (1) is a nonparametric learning algorithm that recursively partitions the feature space into multiple regions and assigns a predicted output to each region. Its hierarchical structure and the discrete nature of the resulting regions make it robust to complex nonlinear relationships and higher-order feature interactions. As a nonparametric method, it also does not require any distributional assumptions to be satisfied; as such, it is well-suited to highly skewed or irregularly distributed data.

In recent years, ensemble learning algorithms based on decision trees, such as Random Forest (2) and Gradient Boosting Machine (3), have gained increasing popularity. First, Random Forest (2) is a bootstrap aggregation method to combine predicted outputs from an ensemble of bagged decision trees, each decision tree built using a random subset of features. Of importance is here that, through random feature selection in the bootstrap aggregation process, Random Forest (2) decorrelates decision trees, thereby accelerating variance reduction and substantially improving predictive accuracy. On the other hand, Gradient Boosting Machine (3), a close competitor to Random Forest (2), refines a predictive model sequentially by fitting weak learners, typically shallow decision trees, to the residuals of prior models. Gradient Boosting Machine (3) shares many advantages of Random Forest (2), yet it learns slowly based on a small learning rate with additional hyperparameters (e.g., maximum tree depth and the number of boosting iterations) to be carefully tuned. Therefore, Random Forest (2) is often faster to train and more automatic in practice.

Random Forest (2) is well-suited to human microbiome studies to predict a host’s health or disease status based on the composition of microbial features (e.g., operational taxonomic units, amplicon sequence variants) in the human body. This is because of the high complexity of microbiome data; the data are highly skewed, zero-inflated, and overdispersed. Here, I pay particular attention to the fact that in human microbiome studies, a phylogenetic tree is often provided as supplementary material alongside microbial feature abundance data. It is constructed based on genetic sequence similarities among microbial features and represents evolutionary ancestry and functional relatedness among microbial features (4). Proper incorporation of phylogenetic tree information into microbiome data analysis has provided new insights and improved analytic performance in α- and β-diversity estimation (5, 6), significance testing (7, 8), causal inference (9, 10), and predictive modeling (11–14).

In this paper, I introduce an extension of the Random Forest (2) algorithm that incorporates phylogenetic tree information, named Phylogeny-Informed Random Forests (PIRF), to improve predictive accuracy in human microbiome studies. Motivating prior extensions of Random Forest are feature elimination (15, 16) and feature weighting (17) approaches designed to address high-dimensional settings by leveraging feature importance scores (e.g., increase in training loss when each feature is permuted or decrease in training loss when each feature is used for splitting). More specifically, feature importance scores have been used either to eliminate less informative features (i.e., to exclude them from the random feature selection process) (15, 16) or to assign higher weights to more informative features (i.e., to increase their likelihood of being included in the random subset of features) (17). These approaches are intuitive and appealing for enhancing the strength of the fitted trees (2) and have demonstrated improved predictive accuracy in several empirical settings (15–17). However, they also entail a potential drawback; that is, when certain features are selected more frequently (i.e., not equally likely), similar features tend to be used across decision trees, leading to an increase in correlation among decision trees. Such an inflated correlation undermines the variance-reduction benefit in the bootstrap aggregation process, ultimately reducing predictive accuracy. As a remedy, PIRF adopts a localized approach; instead of treating all features as competing globally to be selected or weighted, PIRF identifies informative features within each phylogenetic cluster (i.e., a localized group of microbial features that are evolutionarily and functionally related). For this, PIRF first partitions the microbial feature space into multiple phylogenetic clusters using phylogenetic tree information. PIRF then computes feature importance scores within each cluster and converts them into cluster-specific probabilities. Finally, the cluster-specific probabilities are integrated over all phylogenetic clusters to derive community-level selection probabilities. This localized strategy diversifies functional representations while mitigating tree-to-tree correlation.

I demonstrate the high predictive accuracy of PIRF, compared with other off-the-shelf tools, using (i) four classification tasks on gingival inflammation (18), immunotherapy response (19), type 1 diabetes (T1D) (20), and obesity (21) and (ii) three regression tasks on cytokine level (18), age based on oral microbiome (18), and age based on gut microbiome (21). The software and tutorials are freely available as an R package, named PIRF, at https://github.com/hk1785/PIRF. Its core routines are built on the R package ranger (22), which is implemented in C++ and supports multi-core parallel computation. Its trained model can also be easily stored, shared, and applied to new observations for rapid prediction without the need for retraining.

## MATERIALS AND METHODS

### Analytic procedures of PIRF

The analytic procedures of PIRF consist of three main steps: (Step 1) constructing phylogenetic clusters using phylogenetic tree information; (Step 2) computing feature importance scores within each phylogenetic cluster and then converting them into community-level feature selection probabilities; and (Step 3) training a final random forest that selects subsets of features according to the community-level selection probabilities.

Step 1 is described in Phylogenetic Clusters section, and Step 2 is described in Localized Feature Selection and Weighting section. Technical details on tuning the candidate numbers of randomly selected features for computing cluster-specific feature importance scores in Step 2, as well as for training the final random forest in Step 3, together with other technical and implementation details, are provided in ”Other technical details” section.

### Phylogenetic clusters

Microbial features (e.g., operational taxonomic units, amplicon sequence variants) are taxonomic units defined and classified based on genetic sequence similarities, typically using 16S ribosomal RNA genes in amplicon sequencing or genome-wide sequences in shotgun metagenomics (4). In the context of predictive modeling, they are referred to as features or input variables and represented by their measured quantities (typically, relative abundances), denoted as in equation 1.


(1) $$X_{ij}\text{ for }i=1,\ldots ,n\text{ and }j=1,\ldots ,p,$$

where n is the number of subjects and p is the number of features, possibly with the high dimensionality of p≫n.

To characterize the evolutionary and functional relatedness among microbial features, a phylogenetic tree is constructed based on their genetic sequence similarities (4). It consists of leaves (representing microbial features), internal nodes (representing common ancestors), and branches that connect nodes. The length of each branch reflects the evolutionary distance (i.e., the degree of genetic divergence) between nodes. Microbial features with high genetic sequence similarity are considered phylogenetically related, sharing a recent common ancestor and exhibiting similar genetic characteristics and potentially biological functions.

Phylogenetic clusters are localized groups of microbial features that are phylogenetically related. To identify these clusters, PIRF computes pairwise cophenetic distances between microbial features using the phylogenetic tree information (23) and then applies the partitioning around medoids algorithm (24) to partition p features into k clusters. Note that these phylogenetic clusters are constructed algorithmically and do not necessarily correspond to discrete, semantically interpretable biological modules. Here, the cophenetic distance is a phylogenetic distance measure defined as the total branch length connecting two features to their most recent common ancestor (i.e., the nearest internal node) (23). In microbiome data, most microbial features are rare variants with excessive zeros, which makes abundance-based distances ineffective for separating them into biologically meaningful clusters; as such, the cophenetic distance (23) has been widely used for clustering microbial features into distinct functional groups (8, 25).

The number of clusters (k) depends on how the phylogenetic relatedness is defined: (i) the most conservative definition yields fully disjoint clusters (k=p; each feature forms its own cluster), (ii) the most lenient definition yields a single cluster (k=1; all features in one cluster); and (iii) a moderate definition yields 1<k<p. For an optimal clustering resolution, PIRF identifies the number of clusters, denoted as $k_{phylo}$, by maximizing the average silhouette width (i.e., a measure of how well each feature fits within its own cluster relative to others) (26) over the range of 2–10 clusters. Note that the two boundary cases, a single cluster (k=1) and fully disjoint clusters (k=p), are non-phylogenetic clusters, whereas $k_{phylo}$ clusters identified by PIRF are phylogenetic clusters derived from the phylogenetic tree. I denote $k_{phylo}$ phylogenetic clusters as in equation 2.


(2) $$C_{k}=\{j_{1_{k}},\ldots ,j_{q_{k}}\}\text{ for }k=1,\ldots ,k_{phylo},$$

where $q_{k}$ is the number of microbial features in the kth cluster. These clusters are mutually exclusive and exhaustive: $C_{k}\cap C_{k^{\prime }}=\emptyset$ for $k\neq k^{\prime }$ and $\cup_{k=1}^{k_{phylo}}C_{k}=\{1,\ldots ,p\}$.

### Localized feature selection and weighting

Feature importance scores quantify the contribution of each feature to predictive performance. Two notable approaches are (i) permutation-based importance, which measures the increase in training loss when each feature is permuted (randomly shuffled), averaged over multiple permutations and (ii) split-based importance, which measures the decrease in training loss from splits involving each feature, averaged over all bagged trees (27, 28). PIRF adopts permutation-based importance because it is model-agnostic, enabling consistent comparison across different algorithms, less biased to continuous or high-cardinality categorical features, and captures feature interactions more effectively (27, 28).

PIRF computes feature importance scores within each phylogenetic cluster, denoted as in equation 3.


(3) $$\{s_{j_{1_{k}}},\ldots ,s_{j_{q_{k}}}\}\text{ for }k=1,\ldots ,k_{phylo}.$$

A negative feature score indicates that when a feature is permuted (randomly shuffled), predictive performance is improved, indicating no evidence of importance; hence, in equation 3, negative scores are set to zero for feature elimination, while positive scores are retained for feature selection. Then, the cluster-specific importance scores in equation 3 are converted into cluster-specific probabilities as in equation 4.


(4) $$\{v_{j_{1_{k}}},\ldots ,v_{j_{q_{k}}}\}=\{\frac{s_{j_{1_{k}}}}{\sum_{r=1_{k}}^{q_{k}}s_{j_{r}}},\ldots ,\frac{s_{j_{q_{k}}}}{\sum_{r=1_{k}}^{q_{k}}s_{j_{r}}}\}\text{ for }k=1,\ldots ,k_{phylo},$$

where $\sum_{r=1_{k}}^{q_{k}}v_{j_{r}}=1$. Finally, the cluster-specific probabilities in equation 4 are integrated over all phylogenetic clusters to derive community-level selection probabilities as in equation 5.


$$\left\{w_{j_{1_{1}}},\ldots ,w_{j_{q_{1}}},\ldots ,w_{j_{1k_{phylo}}},\ldots ,w_{j_{qk_{phylo}}}\right\}$$

(5) $$=\{v_{j_{1_{1}}}\times (q_{1}/p),\ldots ,v_{j_{q_{1}}}\times (q_{1}/p),\ldots ,v_{j_{1k_{phylo}}}\times (q_{k_{phylo}}/p),\ldots ,v_{j_{qk_{phylo}}}\times (q_{k_{phylo}}/p)\}.$$

Here, each cluster-specific probability is multiplied by the ratio of its cluster size to the total number of features (i.e., if a feature belongs to the kth cluster, its cluster-specific probability is multiplied by $\left(q_{k}/p\right)$). This operation ensures (i) $w_{j_{r}}$’s for $r=1_{k},\ldots ,q_{k}$ and $k=1,\ldots ,k_{phylo}$ to form p probabilities for p features, satisfying the unit-sum constraint of $\sum_{k=1}^{k_{phylo}}\sum_{r=1_{k}}^{q_{k}}w_{j_{r}}=1$ and (ii) every feature to have an equal opportunity of being weighted, regardless of its cluster size. The probabilities in equation 5 are feature-specific weights that quantify the relative importance of microbial features within their localized phylogenetic cluster. These probabilities provide a probabilistic mechanism for selecting subsets of features. That is, in contrast to the standard Random Forest (2), which selects features uniformly at random, PIRF selects features according to the probabilities in equation 5, prioritizing more informative microbial features.

Note that when the localized feature selection and weighting procedures from equations 3 to 5-5 are applied, the two non-phylogenetic clustering cases, a single cluster (k=1) and fully disjoint clusters (k=p), correspond, respectively, to the one-cluster-based feature selection and weighting approach (17) and the standard Random Forest (2).

### Other technical details

In Random Forest (2), the number of randomly selected features is a key tuning parameter. PIRF requires tuning this parameter at two levels: (i) the cluster level, where random forests are trained within each phylogenetic cluster to compute feature importance scores in equation 3 and (ii) the global level, where a final random forest is trained using the community-level selection probabilities in equation 5.

Within each phylogenetic cluster containing $q_{k}$ microbial features ($k=1,\ldots ,k_{phylo}$), I computed cluster-specific feature importance scores in equation 3 by averaging the feature importance scores from three candidate values: $\left\lceil q_{k}\times (1/10)\right\rceil$, $\left\lceil \sqrt{q_{k}}\right\rceil$, and $\left\lceil \log_{2}q_{k}\right\rceil$. At the global level, I considered three analogous candidate values: $\left\lceil p^{\prime }\times (1/10)\right\rceil$, $\left\lceil \sqrt{p^{\prime }}\right\rceil$, and $\left\lceil \log_{2}p^{\prime }\right\rceil$, where $p^{\prime }$ is the number of non-zero probabilities in equation 5, and selected the optimal value by minimizing out-of-bag error rates over 10,000 bagged decision trees.

I set all other hyperparameters to the default settings in the ranger package (22): the minimum node size was 1 for classification and 5 for regression, with node splitting criteria based on Gini impurity for classification and variance reduction for regression (https://cran.r-project.org/web/packages/ranger/index.html). Additional user options are available in the PIRF software package (https://github.com/hk1785/PIRF).

## RESULTS

I evaluated the predictive accuracy of PIRF, relative to other off-the-shelf tools, as follows. For classification tasks, test error rate and test area under the curve (AUC) were employed, while for regression tasks, test root mean squared error (RMSE) and test mean absolute error (MAE) were employed. To estimate these predictive performance metrics, I partitioned the entire data set into fivefold: with fourfold used for training and the remaining onefold used for testing. This process was repeated five times so that each fold was used once for testing. The final performance estimates were obtained by averaging the estimates across the fivefold. In addition, the standard deviation of the estimates across the fivefold was computed as a measure of variability in test performance.

As is standard practice, all feature importance calculations and model training procedures were performed exclusively on the four training folds, while the held-out test fold was used solely for evaluating predictive performance. Details of the microbiome data sets I used and the other off-the-shelf tools I compared are provided in the following sections.

### Microbiome data sets

I considered seven benchmark tasks: (i) four classification problems on gingival inflammation (18), immunotherapy response (19), T1D (20), and obesity (21) and (ii) three regression problems on cytokine level (18), age based on oral microbiome (18), and age based on gut microbiome (21). For all these tasks, quality-control filters were applied uniformly, retaining only the subjects with more than 2,000 total reads and the microbial features with mean relative abundance greater than 0.00001.

To correct for batch effects arising from differences in studies, experimental times, and/or environments, I applied a nonparametric batch correction method, known as conditional quantile regression (ConQuR) (29). ConQuR accounts for zero inflation and distributional heterogeneity in microbiome data while preserving the underlying variability due to other sources, producing batch-effect-free count data suitable for downstream analysis.

Additional preprocessing procedures and the resulting descriptive statistics are as follows.

- **Inflammation** (18): For the gingival inflammation classification task, I used oral microbiome data from the salivary niche of participants in Baltimore, MD (18). The data set included 217 subjects (115 without inflammation; 102 with gingival inflammation) and 2,319 microbial features. The variation due to sample collection times and e-cigarette use was adjusted for using ConQuR (29).
- **Immunotherapy** (19): For the immunotherapy response classification task, I used gut microbiome data on the efficacy of cancer immunotherapy in metastatic melanoma patients, compiled in the meta-analysis of (19). The data set included 257 subjects (168 non-respondents; 89 respondents) and 986 microbial features, collected from five different studies (30–34). The study-specific variation was adjusted for using ConQuR (29).
- **T1D** (20): For the T1D classification task, I used gut microbiome data from the non-obese diabetic mouse model experiment of reference 20. The data set included 521 subjects (167 without T1D; 354 with T1D) and 307 microbial features. The variation due to sample collection times and antibiotic use was adjusted for using ConQuR (29).
- **Obesity** (21): For the obesity classification task, I used gut microbiome data from US-born residents in the American Gut Project (AGP) (21). The data set included 2,162 subjects (1,813 normal; 349 obese) and 2,162 microbial features.
- **Cytokine** (18): For the cytokine level regression task, I used oral microbiome data from the salivary niche of participants in Baltimore, MD (18). Cytokines are small signaling proteins secreted by immune and epithelial cells, playing essential roles in inflammation, immune responses, and intercellular communication. In this analysis, interleukin-8 (IL-8), a prototypical pro-inflammatory cytokine of the chemokine family, was used as a surrogate marker of cytokine level. The data set included 190 subjects (minimum IL-8: 6.66; Q1 IL-8: 237.02; median IL-8: 532.12; Q3 IL-8: 890.49; maximum IL-8: 2359.28) and 2,323 microbial features. The variation due to sample collection times and e-cigarette use was adjusted for using ConQuR (29).
- **Age (Oral)** (18): For the age (oral) regression task, I used oral microbiome data from the salivary niche of participants in Baltimore, MD (18). The data set included 217 subjects (minimum age: 18; Q1 age: 20; median age: 24; Q3 age: 27; maximum age: 34) and 2,319 microbial features. The variation due to sample collection times and e-cigarette use was adjusted for using ConQuR (29).
- **Age (Gut)** (21): I used gut microbiome data from US-born residents in AGP (21). The data set included 2,723 subjects (minimum age: 20; Q1 age: 35; median age: 46; Q3 age: 59; maximum age: 69) and 2,111 microbial features.

### Other off-the-shelf tools

I evaluated 10 other off-the-shelf tools in predictive accuracy as follows.

- **Random Forest** (2): Standard random forest corresponding to fully disjoint clusters (k=p). The same parameter settings and training procedures as in PIRF were applied.
- **Random Forest (One Cluster)** (17): Random forest with one-cluster-based feature selection and weighting (k=1). The same parameter settings and training procedures as in PIRF were applied.
- **Gradient Boosting Machine** (3): Gradient boosting trained with a learning rate of 0.0001, a maximum of 50,000 iterations, and early stopping after 10 rounds. Candidate interaction depths of 1–5 were tuned using 5-fold cross-validation, with cross-entropy loss for classification tasks and mean squared error for regression tasks. All other parameters were set to the defaults of the xgboost package (35) (https://cran.r-project.org/web/packages/xgboost/index.html).
- **Ridge Regression** (36): Ridge regression trained with the regularization parameter tuned using fivefold cross-validation, with cross-entropy loss for classification tasks and mean squared error for regression tasks. All other parameters were set to the defaults of the glmnet package (https://cran.r-project.org/web/packages/glmnet/index.html).
- **Lasso** (37): Lasso regression trained with the regularization parameter tuned using fivefold cross-validation, with cross-entropy loss for classification tasks and mean squared error for regression tasks. All other parameters were set to the defaults of the glmnet package (https://cran.r-project.org/web/packages/glmnet/index.html).
- **Elastic Net** (38): Elastic net regression trained with the regularization and mixing parameters tuned using fivefold cross-validation, with cross-entropy loss for classification tasks and mean squared error for regression tasks. All other parameters were set to the defaults of the glmnet package (https://cran.r-project.org/web/packages/glmnet/index.html).
- S**ingle-layer Neural Network (Keras)** (39, 40): Single-layer neural network trained with the Adam optimizer (41), a learning rate of 0.0001, an early stopping patience of 30, and a maximum of 300 epochs. Candidate numbers of neurons per layer were [p×0.1] and [p×0.2]; candidate dropout rates were 0.3–0.5; candidate batch sizes were [n×0.05] and [n×0.1]; and candidate $L_{2}$ regularization parameters were 0, 10^−6^, and 10^−5^. These hyperparameters were tuned using the fivefold validation-set approach, with cross-entropy loss for classification tasks and mean squared error for regression tasks. All other parameters were set to the defaults of the keras3 package (https://cran.r-project.org/web/packages/keras3/index.html).
- **Single-layer Neural Network (Torch)** (39, 40, 42): Single-layer neural network trained with the Adam optimizer (41), a learning rate of 0.0001, an early stopping patience of 30, and a maximum of 300 epochs. Candidate numbers of neurons per layer were [p×0.1] and [p×0.2]; candidate dropout rates were 0.3–0.5; candidate batch sizes were [n×0.05] and [n×0.1]; and candidate $L_{2}$ regularization parameters were 0, 10^−6^, and 10^−5^. These hyperparameters were tuned using the fivefold validation-set approach, with cross-entropy loss for classification tasks and mean squared error for regression tasks. All other parameters were set to the defaults of the torch package (42) (https://cran.r-project.org/web/packages/torch/index.html).
- **Deep Neural Network (Keras)** (39, 40): Two-layer neural network trained with the Adam optimizer (41), a learning rate of 0.0001, an early stopping patience of 30, and a maximum of 300 epochs. Candidate numbers of neurons per layer were [p×0.1] and [p×0.2]; candidate dropout rates were 0.3–0.5; candidate batch sizes were [n×0.05] and [n×0.1]; and candidate $L_{2}$ regularization parameters were 0, 10^−6^, and 10^−5^. These hyperparameters were tuned using the fivefold validation-set approach, with cross-entropy loss for classification tasks and mean squared error for regression tasks. All other parameters were set to the defaults of the keras3 package (https://cran.r-project.org/web/packages/keras3/index.html).
- **Deep Neural Network (Torch)** (39, 40, 42): Two-layer neural network trained with the Adam optimizer (41), a learning rate of 0.0001, an early stopping patience of 30, and a maximum of 300 epochs. Candidate numbers of neurons per layer were [p×0.1] and [p×0.2]; candidate dropout rates were 0.3–0.5; candidate batch sizes were [n×0.05] and [n×0.1]; and candidate $L_{2}$ regularization parameters were 0, 10^−6^, and 10^−5^. These hyperparameters were tuned using the fivefold validation-set approach, with cross-entropy loss for classification tasks and mean squared error for regression tasks. All other parameters were set to the defaults of the torch package (42) (https://cran.r-project.org/web/packages/torch/index.html).

### Predictive performances

The predictive performances across different tasks and methods are organized as follows.

- Table 1 reports the test error rates and AUC values for the four classification tasks: Inflammation (18), Immunotherapy (19), T1D (20), and Obesity (21) using each of the 11 tools: PIRF, Random Forest (2), Random Forest (One Cluster) (17), Gradient Boosting Machine (3), Ridge Regression (36), Lasso (37), Elastic Net (38), Single-layer Neural Network (Keras) (39, 40), Single-layer Neural Network (Torch) (39, 40, 42), Deep Neural Network (Keras) (39, 40), and Deep Neural Network (Torch) (39, 40, 42).
- Table 2 reports the test RMSE and MAE values for the three regression tasks: Cytokine (18), Age (Oral) (18), and Age (Gut) (21) using each of the 11 tools: PIRF, Random Forest (2), Random Forest (One Cluster) (17), Gradient Boosting Machine (3), Ridge Regression (36), Lasso (37), Elastic Net (38), Single-layer Neural Network (Keras) (39, 40), Single-layer Neural Network (Torch) (39, 40, 42), Deep Neural Network (Keras) (39, 40), and Deep Neural Network (Torch) (39, 40, 42).

**TABLE 1:** The average test error rates and AUC values across the fivefold (unit: %), with standard deviations shown in parentheses, for PIRF and other existing off-the-shelf tools for the classification tasks of Inflammation, Immunotherapy, T1D, and Obesity^*a*^

	Inflammation *n* = 217 *p* = 2,319	Immunotherapy *n* = 257 *p* = 986	T1D *n* = 521 p = 307	Obesity *n* = 2,162 *p* = 2,162
Test Error Rate				
PIRF	10.20 (6.42)	8.15 (4.17)	11.13 (1.60)	15.91 (0.20)
RF	11.57 (6.42)	8.93 (2.91)	13.42 (3.40)	16.18 (0.20)
RF (One Cluster)	11.57 (6.91)	8.55 (4.00)	11.32 (1.59)	16.42 (0.21)
GBM	15.26 (7.33)	9.72 (3.04)	14.96 (3.13)	16.60 (1.62)
Ridge	40.53 (7.60)	29.93 (8.66)	20.91 (3.62)	16.48 (1.40)
Lasso	11.62 (3.59)	13.53 (14.29)	23.79 (7.08)	16.63 (1.65)
EN	12.07 (4.20)	19.01 (15.74)	22.83 (6.31)	16.58 (1.49)
SNN (Keras)	30.84 (3.88)	35.83 (7.53)	31.86 (4.76)	16.24 (1.43)
SNN (Torch)	36.43 (10.62)	21.41 (8.25)	32.04 (4.47)	16.10 (1.14)
DNN (Keras)	33.59 (5.41)	34.66 (7.06)	31.28 (4.63)	16.25 (1.45)
DNN (Torch)	42.81 (7.60)	16.38 (5.74)	32.04 (4.47)	16.15 (1.26)
Test AUC				
PIRF	96.84 (3.47)	94.20 (2.97)	94.95 (1.43)	74.05 (0.23)
RF	96.17 (3.12)	91.77 (4.71)	93.51 (1.87)	71.57 (0.23)
RF (One Cluster)	96.21 (3.65)	93.14 (2.71)	94.74 (1.40)	73.89 (0.24)
GBM	92.27 (5.34)	87.00 (5.88)	93.28 (2.56)	66.42 (5.65)
Ridge	79.49 (8.65)	87.31 (8.39)	79.61 (3.78)	70.98 (2.55)
Lasso	94.20 (3.25)	82.26 (19.13)	79.24 (5.68)	68.06 (3.00)
EN	94.92 (2.91)	75.75 (23.92)	80.08 (3.75)	72.01 (4.92)
SNN (Keras)	78.68 (4.11)	59.77 (3.27)	59.26 (2.93)	53.99 (2.71)
SNN (Torch)	70.11 (17.15)	87.44 (1.53)	56.46 (6.74)	66.40 (6.40)
DNN (Keras)	78.11 (6.26)	56.85 (6.97)	62.21 (4.26)	53.52 (2.52)
DNN (Torch)	63.85 (7.28)	89.32 (2.93)	56.18 (4.27)	69.01 (3.32)

^*a*^ RF, RF (One Cluster), GBM, Ridge, Lasso, EN, SNN (Keras), SNN (Torch), DNN (Keras), and DNN (Torch) represent Random Forest, Random Forest (One Cluster), Gradient Boosting Machine, Ridge Regression, Lasso, Elastic Net, Single-layer Neural Network (Keras), Single-layer Neural Network (Torch), Deep Neural Network (Keras), and Deep Neural Network (Torch), respectively.

**TABLE 2:** The average test RMSE and MAE values across the fivefold, with standard deviations shown in parentheses, for PIRF and other existing off-the-shelf tools for the regression tasks of Cytokine, Age (Oral), and Age (Gut)^*a*^

	Cytokine *n* = 190 *p* = 2,323	Age (Oral) *n* = 217 *p* = 2,319	Age (Gut) *n* = 2,723 *p* = 2,111
Test RMSE			
PIRF	241.20 (32.14)	2.20 (0.29)	11.53 (0.20)
RF	259.05 (27.97)	2.36 (0.22)	11.69 (0.20)
RF (One Cluster)	244.85 (32.89)	2.31 (0.31)	11.79 (0.21)
GBM	277.42 (40.36)	3.36 (0.22)	12.52 (0.16)
Ridge	523.76 (61.21)	5.21 (2.34)	14.19 (0.29)
Lasso	441.05 (32.30)	3.93 (0.30)	13.03 (0.37)
EN	404.24 (49.88)	5.20 (3.38)	13.34 (1.24)
SNN (Keras)	650.69 (44.53)	4.23 (0.23)	13.30 (0.23)
SNN (Torch)	640.17 (43.94)	4.19 (0.26)	13.62 (0.28)
DNN (Keras)	471.02 (40.25)	4.14 (0.22)	16.92 (0.41)
DNN (Torch)	452.44 (45.55)	4.12 (0.20)	14.15 (0.25)
Test MAE			
PIRF	144.18 (30.23)	1.35 (0.14)	9.37 (0.23)
RF	169.81 (21.91)	1.56 (0.09)	9.50 (0.23)
RF (One Cluster)	147.29 (30.28)	1.47 (0.16)	9.65 (0.24)
GBM	182.50 (23.04)	2.60 (0.20)	10.26 (0.14)
Ridge	385.15 (35.89)	3.89 (0.94)	12.09 (0.25)
Lasso	363.90 (15.45)	3.30 (0.30)	10.09 (0.23)
EN	328.73 (31.46)	3.42 (0.80)	10.70 (0.29)
SNN (Keras)	487.91 (26.56)	3.51 (0.18)	11.27 (0.24)
SNN (Torch)	478.62 (27.69)	3.53 (0.14)	11.74 (0.26)
DNN (Keras)	369.19 (24.22)	3.42 (0.19)	13.64 (0.48)
DNN (Torch)	370.16 (23.40)	3.49 (0.11)	12.23 (0.26)

^*a*^ RF, RF (One Cluster), GBM, Ridge, Lasso, EN, SNN (Keras), SNN (Torch), DNN (Keras), and DNN (Torch) represent Random Forest, Random Forest (One Cluster), Gradient Boosting Machine, Ridge Regression, Lasso, Elastic Net, Single-layer Neural Network (Keras), Single-layer Neural Network (Torch), Deep Neural Network (Keras), and Deep Neural Network (Torch), respectively.

To summarize, PIRF showed the strongest predictive performance across all tasks: for classification, it achieved the lowest test error rate and the highest test AUC (Table 1); for regression, it achieved the lowest test RMSE and the lowest test MAE (Table 2).

Among the comparison methods, tree-based models—including Random Forest (2), Random Forest (One Cluster) (17), and Gradient Boosting Machine (35)—generally outperformed linear models—including Ridge Regression (36), Lasso (37), and Elastic Net (38)—for most tasks (Tables 1 and 2), which is likely due to their ability to capture nonlinear relationships and higher-order feature interactions. Note also that the recently popular Deep Neural Networks (39, 40) implemented in Keras and Torch (42) consistently showed the weakest predictive performance (Tables 1 and 2), which is likely due to the p≫n regime, typical of microbiome data; the high dimensionality combined with relatively small to moderate sample sizes ($n\approx 10^{2}∼10^{3}$), which elevates overfitting risk for high-capacity models whose benefits usually emerge only with much larger samples (e.g., $n>10^{5}$). In addition, Single-layer Neural Networks (39, 40) do not exhibit systematic performance improvements over their deeper counterparts, suggesting that architectural depth alone does not alleviate the fundamental sample size limitations in this context.

With respect to performance variability, PIRF exhibits stable performance with no notable inflation in standard deviations across tasks (Tables 1 and 2). In contrast, a few competing methods show substantial variability, including Ridge Regression (36) for the Cytokine (18) and Age (Oral) (18) tasks (Table 2); Lasso (37) and Elastic Net (38) for the Immunotherapy (19) task (Table 1); Elastic Net (38) additionally for the Age (Oral) (18) task (Table 2); and both Single-layer Neural Networks and Deep Neural Networks (39, 40) implemented in Torch (42) for the Inflammation (18) and Obesity (21) tasks (Table 1). These findings indicate reduced robustness of these methods under repeated data splits.

### Probabilistic mechanism of PIRF

**Fig 1:**
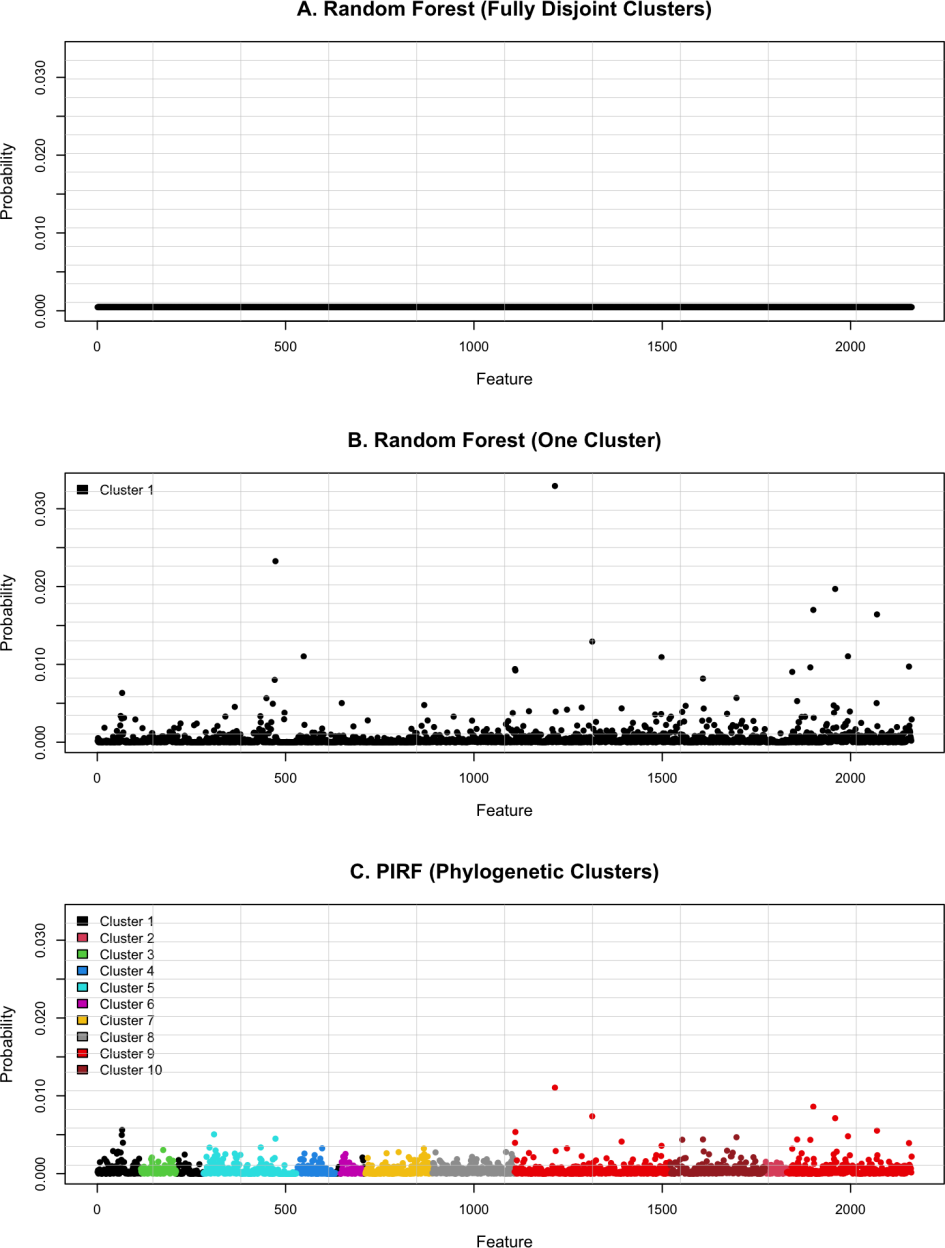
Visual representation of the selection probabilities for the classification task of Obesity using three methods: (**A**) Random Forest (Fully Disjoint Clusters); (**B**) Random Forest (One Cluster) and (**C**) PIRF (Phylogenetic Clusters). Within each method, the probabilities sum to one; as such, Random Forest (Fully Disjoint Clusters) shows the least variability, Random Forest (One Cluster) shows the highest variability, and PIRF (Phylogenetic Clusters) shows an intermediate level of variability.

To illustrate the probabilistic mechanism of PIRF, relative to the other Random Forest extensions: Random Forest (2) and Random Forest (One Cluster) (17), I created a plot displaying the probabilities of microbial features to be selected by each method. To save space, only the plot for the classification task of Obesity (21) is reported in the main text (Fig. 1), while the ones for the remaining tasks are provided in the Supplemental Materials: Inflammation (18) (Fig. S1); Immunotherapy (19) (Fig. S2); T1D (20) (Fig. S3); Cytokine (18) (Fig. S4); Age (Oral) (18) (Fig. S5); and Age (Gut) (21) (Fig. S6).

Note that for each method, the probabilities sum to one. The probabilistic mechanisms are described as follows.

- **Random Forest (Fully Disjoint Clusters)** (2) (Fig. 1A; Fig. S1A to S6A): Standard Random Forest (2)—which selects features uniformly at random corresponding to the case of fully disjoint clusters (k=p)—yields the lowest variance in probabilities and, thus, maximizes tree decorrelation but lacks any feature selection or weighting mechanism.
- **Random Forest (One Cluster)** (17) (Fig. 1B; Fig. S1B to S6B): Random Forest based on one community-level cluster—which performs feature selection and weighting within a single cluster (k=1)—yields the highest variance in probabilities and, thus, minimizes tree decorrelation and enforces a globalized feature selection and weighting mechanism.
- **PIRF (Phylogenetic Clusters)** (Fig. 1C; Fig. S1C to S6C): Localized Random Forest based on phylogenetic clusters yields intermediate levels of variability in probabilities and tree decorrelation. Importantly, phylogenetic clusters are algorithmically constructed but are grounded in phylogenetic proximity, providing localized neighborhoods of evolutionarily related features that help diversify representations.

To summarize, the standard Random Forest (2) benefits from maximizing tree decorrelation but lacks any mechanism for feature selection and weighting, whereas the Random Forest (One Cluster) (17) emphasizes feature selection and weighting at the expense of substantially reduced tree decorrelation. In contrast, PIRF introduces a localized approach that balances tree decorrelation with feature selection and weighting, while leveraging phylogenetic clusters to diversify functional representations. This balance, coupled with its enriched functional representations, underpins the superior predictive performance of PIRF in microbiome applications.

## DISCUSSION

In this paper, I introduced PIRF, a method designed to improve predictive accuracy in human microbiome studies. The core mechanism of PIRF lies in its localized approach: rather than treating all features as competing globally to be selected or weighted (17), PIRF identifies informative features within each phylogenetic cluster—a localized group of evolutionarily and functionally related microbial features. This strategy enriches functional representations while reducing tree-to-tree correlation. To achieve it, PIRF partitions the microbial feature space into multiple phylogenetic clusters using phylogenetic tree information. It then computes feature importance scores within each cluster and converts them into cluster-specific probabilities. Finally, these cluster-specific probabilities are integrated across all phylogenetic clusters to derive community-level selection probabilities.

To evaluate predictive performance, I applied PIRF to seven benchmark tasks, comprising four classification problems (Inflammation [18], Immunotherapy [19], T1D [20], and Obesity [21]) and three regression problems (Cytokine [18], Age [Oral] [18], and Age [Gut] [21]). Across these tasks, PIRF achieved the strongest predictive performance—yielding the lowest test error rate and the highest test AUC for classification, and the lowest test RMSE and MAE for regression—outperforming other off-the-shelf tools: Random Forest (2), Random Forest (One Cluster) (17), Gradient Boosting Machine (3), Ridge Regression (36), Lasso (37), Elastic Net (38), and two Deep Neural Network implementations (Keras [39, 40] and Torch [39, 40, 42]).

The software and tutorials are freely available as an R package, PIRF, at https://github.com/hk1785/PIRF, whereas many recent methods lack accessible software, limiting their practical utility. PIRF can serve as a useful tool for microbiome-based disease diagnostics and personalized medicine.

## SUPPLEMENTAL MATERIAL

Supplemental material spectrum.03451-25-s0001.pdf Figures S1 to S6.

ASM does not own the copyrights to Supplemental Material that may be linked to, or accessed through, an article. The authors have granted ASM a non-exclusive, world-wide license to publish the Supplemental Material files. Please contact the corresponding author directly for reuse.

## References

[1] Breiman L, Friedman JH, Stone CJ. 1984. Classification and regression trees. CRC Press, Boca Raton, FL, USA.

[2] Breiman L. 2001. Random forests. Mach Learn 45:5–32. doi:10.1023/A:1010933404324

[5] Friedman JH. 2001. Greedy function approximation: a gradient boosting machine. Ann Statist 29. doi:10.1214/aos/1013203451

[4] Gilbert JA, Blaser MJ, Caporaso JG, Jansson JK, Lynch SV, Knight R. 2018. Current understanding of the human microbiome. Nat Med 24:392–400. doi:10.1038/nm.4517 29634682 PMC7043356

[5] Faith DP. 1992. Conservation evaluation and phylogenetic diversity. Biol Conserv 61:1–10. doi:10.1016/0006-3207(92)91201-3

[6] Lozupone C, Knight R. 2005. UniFrac: a new phylogenetic method for comparing microbial communities. Appl Environ Microbiol 71:8228–8235. doi:10.1128/AEM.71.12.8228-8235.2005 16332807 PMC1317376

[7] Anderson MJ. 2001. A new method for non-parametric multivariate analysis of variance. Austral Ecol 26:32–46. doi:10.1046/j.1442-9993.2001.01070.x

[8] Zhao N, Chen J, Carroll IM, Ringel-Kulka T, Epstein MP, Zhou H, Zhou JJ, Ringel Y, Li H, Wu MC. 2015. Testing in microbiome-profiling studies with MiRKAT, the microbiome regression-based kernel association test. Am J Hum Genet 96:797–807. doi:10.1016/j.ajhg.2015.04.003 25957468 PMC4570290

[9] Zhang J, Wei Z, Chen J. 2018. A distance-based approach for testing the mediation effect of the human microbiome. Bioinformatics 34:1875–1883. doi:10.1093/bioinformatics/bty014 29346509

[10] Yue Y, Hu YJ. 2022. A new approach to testing mediation of the microbiome at both the community and individual taxon levels. Bioinformatics 38:3173–3180. doi:10.1093/bioinformatics/btac310 35512399 PMC9191207

[11] Wang Y, Bhattacharya T, Jiang Y, Qin X, Wang Y, Liu Y, Saykin AJ, Chen L. 2021. A novel deep learning method for predictive modeling of microbiome data. Brief Bioinformatics 22:bbaa073. doi:10.1093/bib/bbaa073 32406914 PMC13017428

[12] Koh H. 2023. Subgroup identification using virtual twins for human microbiome studies. IEEE/ACM Trans Comput Biol Bioinform 20:3800–3808. doi:10.1109/TCBB.2023.3324139 37831574

[13] Li B, Wang T, Qian M, Wang S. 2023. MKMR: a multi-kernel machine regression model to predict health outcomes using human microbiome data. Brief Bioinformatics 24:bbad158. doi:10.1093/bib/bbad158 37099694 PMC13017232

[14] Xu H, Wang T, Miao Y, Qian M, Yang Y, Wang S. 2024. MK-BMC: a multi-kernel framework with boosted distance metrics for microbiome data for classification. Bioinformatics 40:btad757. doi:10.1093/bioinformatics/btad757 38200571 PMC10789312

[15] Granitto PM, Furlanello C, Biasioli F, Gasperi F. 2006. Recursive feature elimination with random forest for PTR-MS analysis of agroindustrial products. Chemometr Intell Lab Syst 83:83–90. doi:10.1016/j.chemolab.2006.01.007

[16] Darst BF, Malecki KC, Engelman CD. 2018. Using recursive feature elimination in random forest to account for correlated variables in high dimensional data. BMC Genet 19:65. doi:10.1186/s12863-018-0633-8 30255764 PMC6157185

[17] Liu Y, Zhao H. 2017. Variable importance-weighted Random Forests. Quant Biol 5:338–351. doi:10.1007/s40484-017-0121-6 30034909 PMC6051549

[18] Park B, Koh H, Patatanian M, Reyes-Caballero H, Zhao N, Meinert J, Holbrook JT, Leinbach LI, Biswal S. 2023. The mediating roles of the oral microbiome in saliva and subgingival sites between e-cigarette smoking and gingival inflammation. BMC Microbiol 23:35. doi:10.1186/s12866-023-02779-z 36732713 PMC9893987

[19] Limeta A, Ji B, Levin M, Gatto F, Nielsen J. 2020. Meta-analysis of the gut microbiota in predicting response to cancer immunotherapy in metastatic melanoma. JCI Insight 5:e140940. doi:10.1172/jci.insight.140940 33268597 PMC7714408

[20] Zhang X-S, Li J, Krautkramer KA, Badri M, Battaglia T, Borbet TC, Koh H, Ng S, Sibley RA, Li Y, et al.. 2018. Antibiotic-induced acceleration of type 1 diabetes alters maturation of innate intestinal immunity. eLife 7:1–37. doi:10.7554/eLife.37816 PMC6085123 30039798

[21] McDonald D, Hyde E, Debelius JW, Morton JT, Gonzalez A, Ackermann G, Aksenov AA, Behsaz B, Brennan C, Chen Y, et al.. 2018. American gut: an open platform for citizen science microbiome research. mSystems 3:e00031-18. doi:10.1128/mSystems.00031-18 29795809 PMC5954204

[22] Wright MN, Ziegler A. 2017. ranger: a fast implementation of random forests for high dimensional data in C++ and R. J Stat Softw 77:1–17. doi:10.18637/jss.v077.i01

[23] Schlee D, Sneath PHA, Sokal RR, Freeman WH. 1975. Numerical taxonomy. The principles and practice of numerical classification. Syst Zool 24:263. doi:10.2307/2412767

[24] Reynolds AP, Richards G, de la Iglesia B, Rayward-Smith VJ. 2006. Clustering rules: a comparison of partitioning and hierarchical clustering algorithms. J Math Model Algor 5:475–504. doi:10.1007/s10852-005-9022-1

[25] Koh H, Zhao N. 2020. A powerful microbial group association test based on the higher criticism analysis for sparse microbial association signals. Microbiome 8:63. doi:10.1186/s40168-020-00834-9 32393397 PMC7216722

[26] Rousseeuw PJ. 1987. Silhouettes: a graphical aid to the interpretation and validation of cluster analysis. J Comput Appl Math 20:53–65. doi:10.1016/0377-0427(87)90125-7

[27] Altmann A, Toloşi L, Sander O, Lengauer T. 2010. Permutation importance: a corrected feature importance measure. Bioinformatics 26:1340–1347. doi:10.1093/bioinformatics/btq134 20385727

[28] Louppe G, Wehenkel L, Sutera A, Geurts P. 2013. Understanding variable importances in forests of randomized trees. *In* Advances in Neural Information Processing Systems (NIPS). Vol. 26.

[29] Ling W, Lu J, Zhao N, Lulla A, Plantinga AM, Fu W, Zhang A, Liu H, Song H, Li Z, Chen J, Randolph TW, Koay WLA, White JR, Launer LJ, Fodor AA, Meyer KA, Wu MC. 2022. Batch effects removal for microbiome data via conditional quantile regression. Nat Commun 13:5418. doi:10.1038/s41467-022-33071-9 36109499 PMC9477887

[30] Matson V, Fessler J, Bao R, Chongsuwat T, Zha Y, Alegre ML, Luke JJ, Gajewski TF. 2018. The commensal microbiome is associated with anti–PD-1 efficacy in metastatic melanoma patients. Science 359:104–108. doi:10.1126/science.aao3290 29302014 PMC6707353

[31] Frankel AE, Coughlin LA, Kim J, Froehlich TW, Xie Y, Frenkel EP, Koh AY. 2017. Metagenomic shotgun sequencing and unbiased metabolomic profiling identify specific human gut microbiota and metabolites associated with immune checkpoint therapy efficacy in melanoma patients. Neoplasia 19:848–855. doi:10.1016/j.neo.2017.08.004 28923537 PMC5602478

[32] Gopalakrishnan V, Spencer CN, Nezi L, Reuben A, Andrews MC, Karpinets TV, Prieto PA, Vicente D, Hoffman K, Wei SC, et al.. 2018. Gut microbiome modulates response to anti–PD-1 immunotherapy in melanoma patients. Science 359:97–103. doi:10.1126/science.aan4236 29097493 PMC5827966

[33] Routy B, Le Chatelier E, Derosa L, Duong CPM, Alou MT, Daillère R, Fluckiger A, Messaoudene M, Rauber C, Roberti MP, et al.. 2018. Gut microbiome influences efficacy of PD-1–based immunotherapy against epithelial tumors. Science 359:91–97. doi:10.1126/science.aan3706 29097494

[34] Peters BA, Wilson M, Moran U, Pavlick A, Izsak A, Wechter T, Weber JS, Osman I, Ahn J. 2019. Relating the gut metagenome and metatranscriptome to immunotherapy responses in melanoma patients. Genome Med 11:61. doi:10.1186/s13073-019-0672-4 31597568 PMC6785875

[35] Chen T, Guestrin C. 2016. Xgboost: a scalable tree boosting system. Proceedings of the 22nd ACM SIGKDD International Conference on Knowledge Discovery and Data Mining (KDD). p 785–794. doi:10.1145/2939672.2939785

[36] Hoerl AE, Kennard RW. 1970. Ridge regression: biased estimation for nonorthogonal problems. Technometrics 12:55–67. doi:10.1080/00401706.1970.10488634

[37] Tibshirani R. 1996. Regression shrinkage and selection via the lasso. J R Stat Soc Ser B 58:267–288. doi:10.1111/j.2517-6161.1996.tb02080.x

[38] Zou H, Hastie T. 2005. Regularization and variable selection via the elastic net. J R Stat Soc B 67:301–320. doi:10.1111/j.1467-9868.2005.00503.x

[39] Hinton GE, Osindero S, Teh YW. 2006. A fast learning algorithm for deep belief nets. Neural Comput 18:1527–1554. doi:10.1162/neco.2006.18.7.1527 16764513

[40] LeCun Y, Bengio Y, Hinton G. 2015. Deep learning. Nature 521:436–444. doi:10.1038/nature14539 26017442

[41] Kingma DP, Ba J. 2015. Adam: a method for stochastic optimization. Proceedings of the 3rd International Conference on Learning Representations (ICLR.

[42] Paszke A, Gross S, Massa F, Lerer A, Bradbury J, Chanan G, Killeen T, Lin Z, Gimelshein N, Antiga L, Desmaison A, Kopf A, Yang E, DeVito Z, Raison M, Tejani A, Chilamkurthy S, Steiner B, Fang L, Bai J, Chintala S. 2019. PyTorch: an imperative style, high-performance deep learning library, p 8024–8035. *In* Advances in Neural Information Processing Systems (NeurIPS)

