# Supplementary material for: Phylogeny-informed random forests for human microbiome studies
Source: Microbiol Spectr. 2026 Mar 24;14(5):e03451-25. doi: 10.1128/spectrum.03451-25 (PMC13142014; doi:10.1128/spectrum.03451-25)

S1 Fig. Visual representation of the selection probabilities for the classification task of Inflammation using three methods: A. Random Forest (Fully Disjoint Clusters); B. Random Forest (One Cluster); and C. PIRF (Phylogenetic Clusters). Within each method, the probabilities sum to one; as such, Random Forest (Fully Disjoint Clusters) shows the least variability, Random Forest (One Cluster) shows the highest variability, and PIRF (Phylogenetic Clusters) shows an intermediate level of variability.

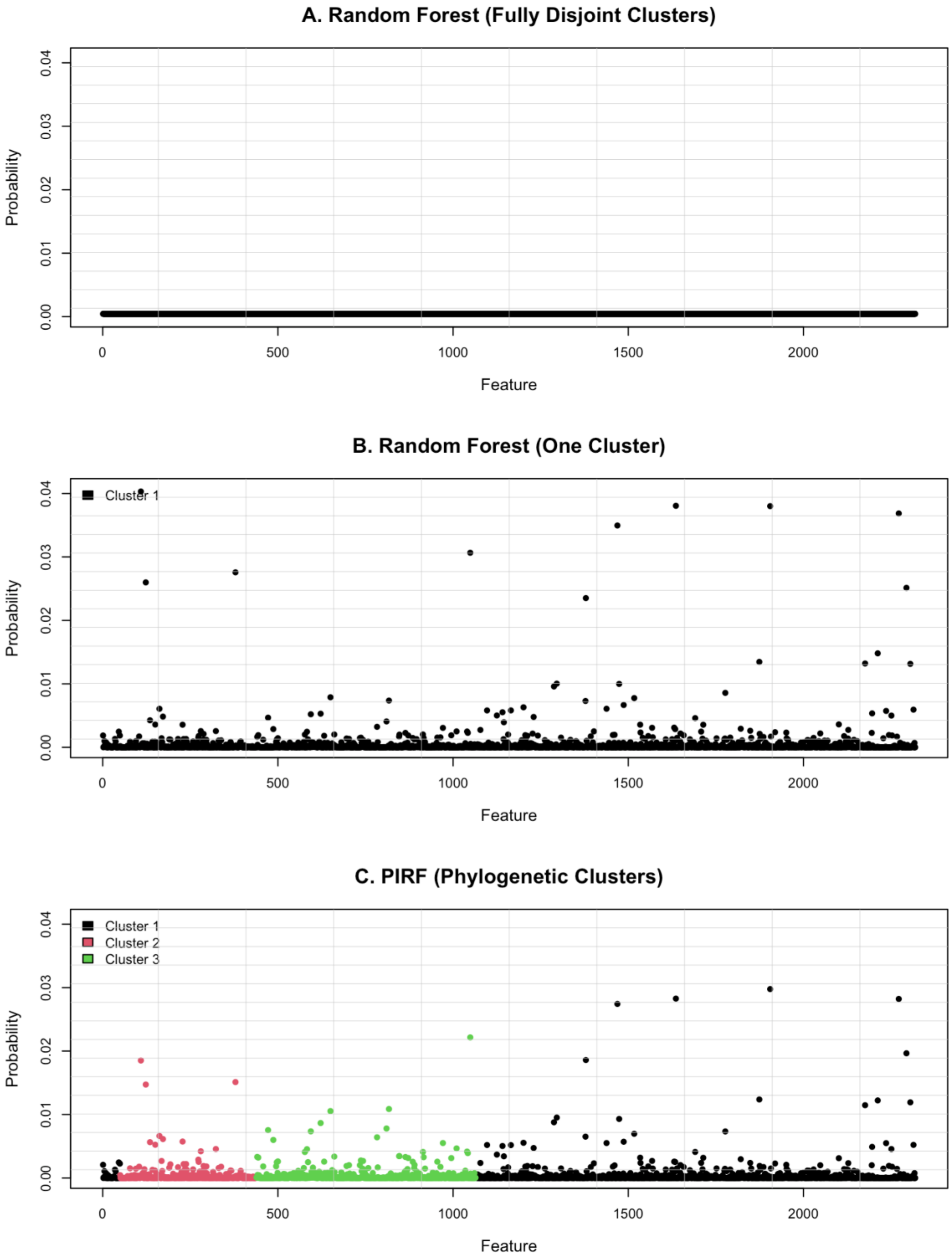

S2 Fig. Visual representation of the selection probabilities for the classification task of Immunotherapy using three methods: A. Random Forest (Fully Disjoint Clusters); B. Random Forest (One Cluster); and C. PIRF (Phylogenetic Clusters). Within each method, the probabilities sum to one; as such, Random Forest (Fully Disjoint Clusters) shows the least variability, Random Forest (One Cluster) shows the highest variability, and PIRF (Phylogenetic Clusters) shows an intermediate level of variability.

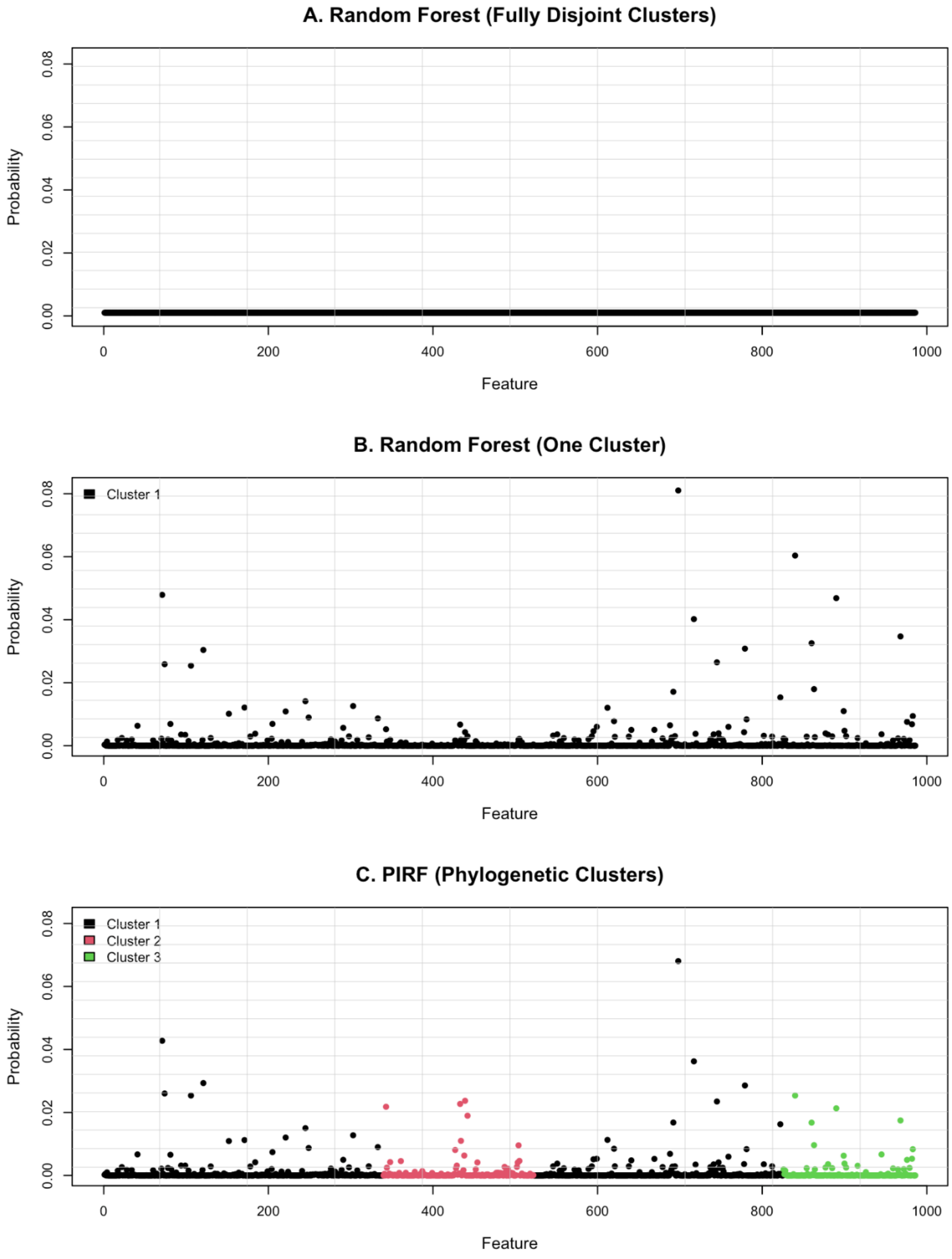

S3 Fig. Visual representation of the selection probabilities for the classification task of T1D using three methods: A. Random Forest (Fully Disjoint Clusters); B. Random Forest (One Cluster); and C. PIRF (Phylogenetic Clusters). Within each method, the probabilities sum to one; as such, Random Forest (Fully Disjoint Clusters) shows the least variability, Random Forest (One Cluster) shows the highest variability, and PIRF (Phylogenetic Clusters) shows an intermediate level of variability.

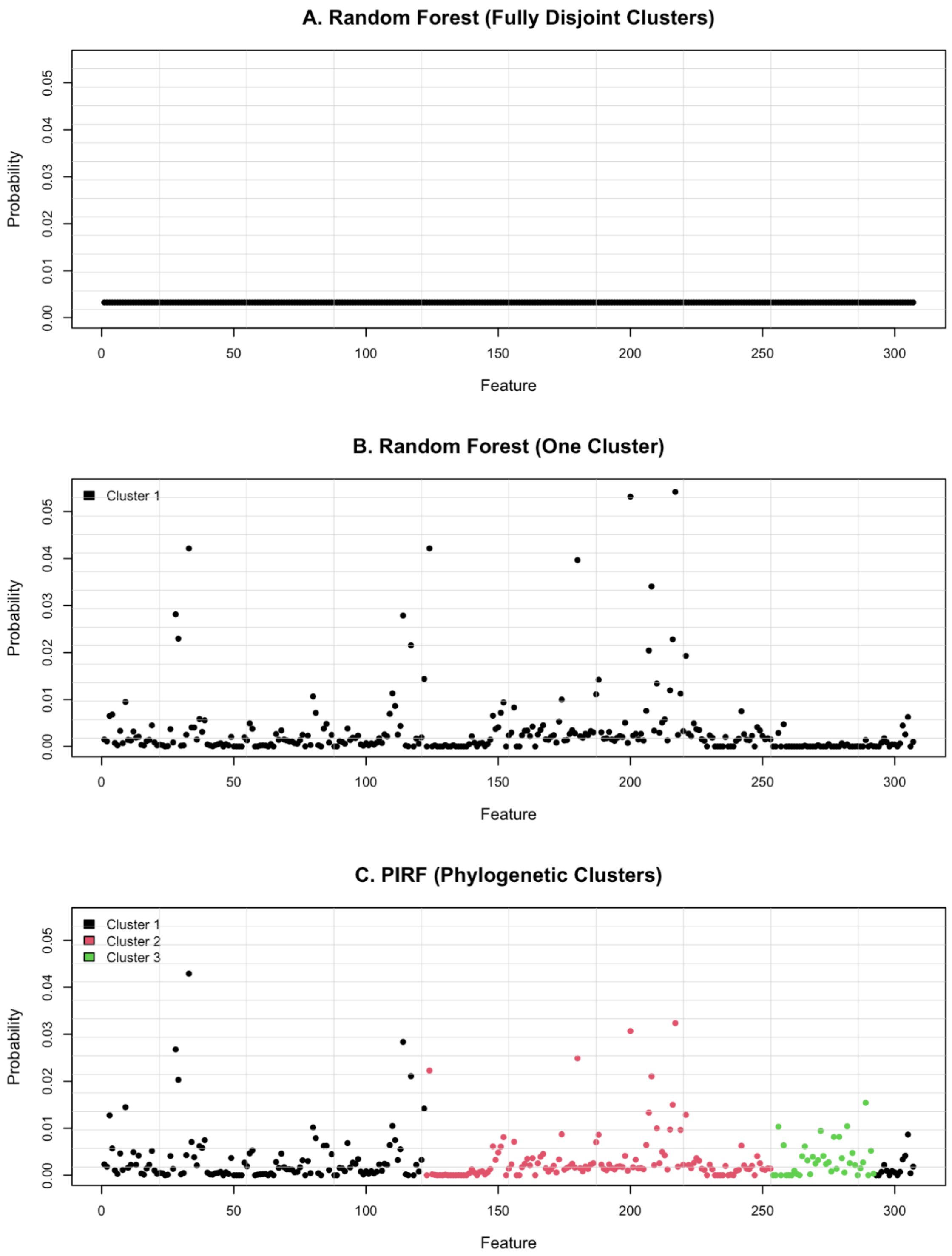

S4 Fig. Visual representation of the selection probabilities for the regression task of Cytokine using three methods: A. Random Forest (Fully Disjoint Clusters); B. Random Forest (One Cluster); and C. PIRF (Phylogenetic Clusters). Within each method, the probabilities sum to one; as such, Random Forest (Fully Disjoint Clusters) shows the least variability, Random Forest (One Cluster) shows the highest variability, and PIRF (Phylogenetic Clusters) shows an intermediate level of variability.

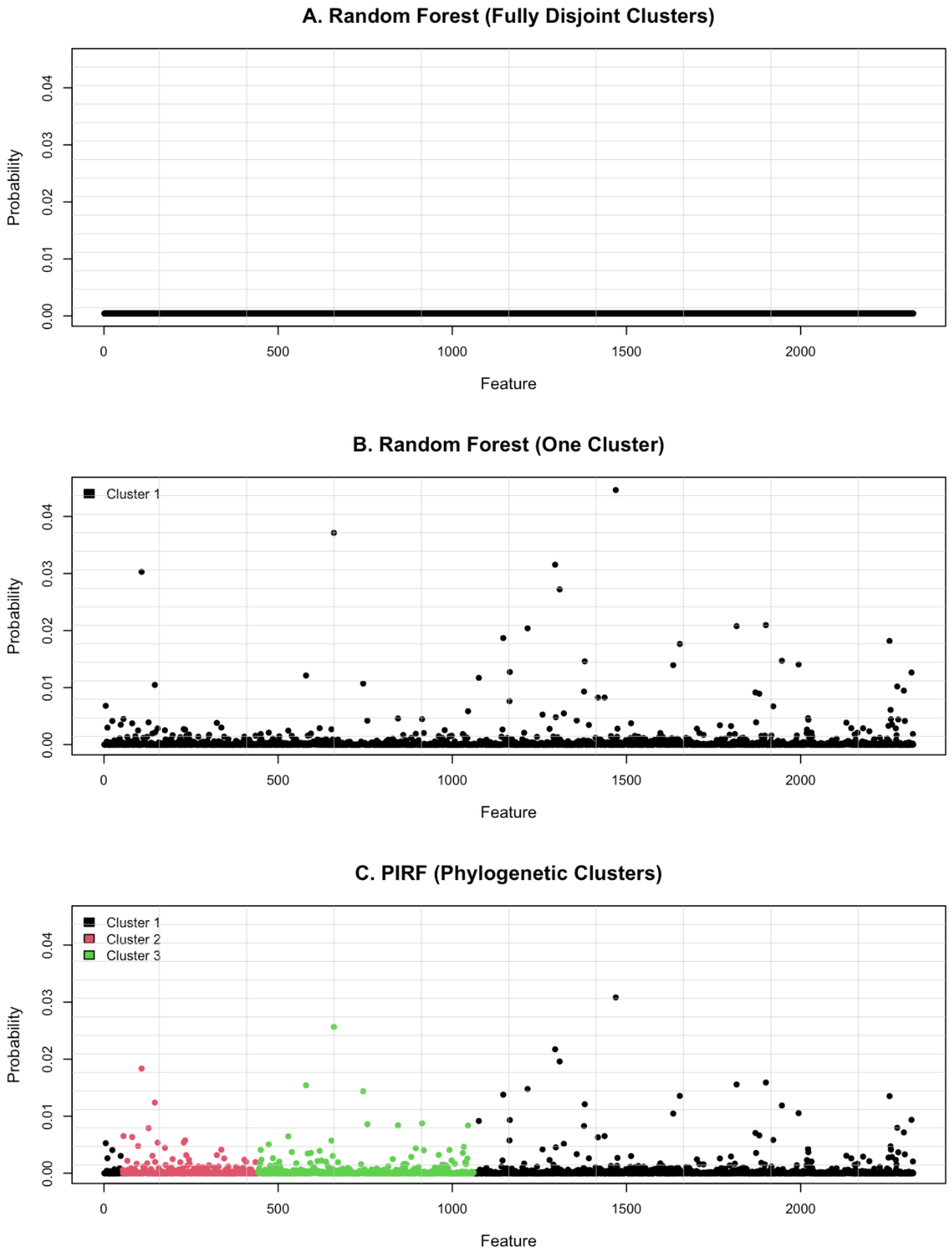

S5 Fig. Visual representation of the selection probabilities for the regression task of Age (Oral) using three methods: A. Random Forest (Fully Disjoint Clusters); B. Random Forest (One Cluster); and C. PIRF (Phylogenetic Clusters). Within each method, the probabilities sum to one; as such, Random Forest (Fully Disjoint Clusters) shows the least variability, Random Forest (One Cluster) shows the highest variability, and PIRF (Phylogenetic Clusters) shows an intermediate level of variability.

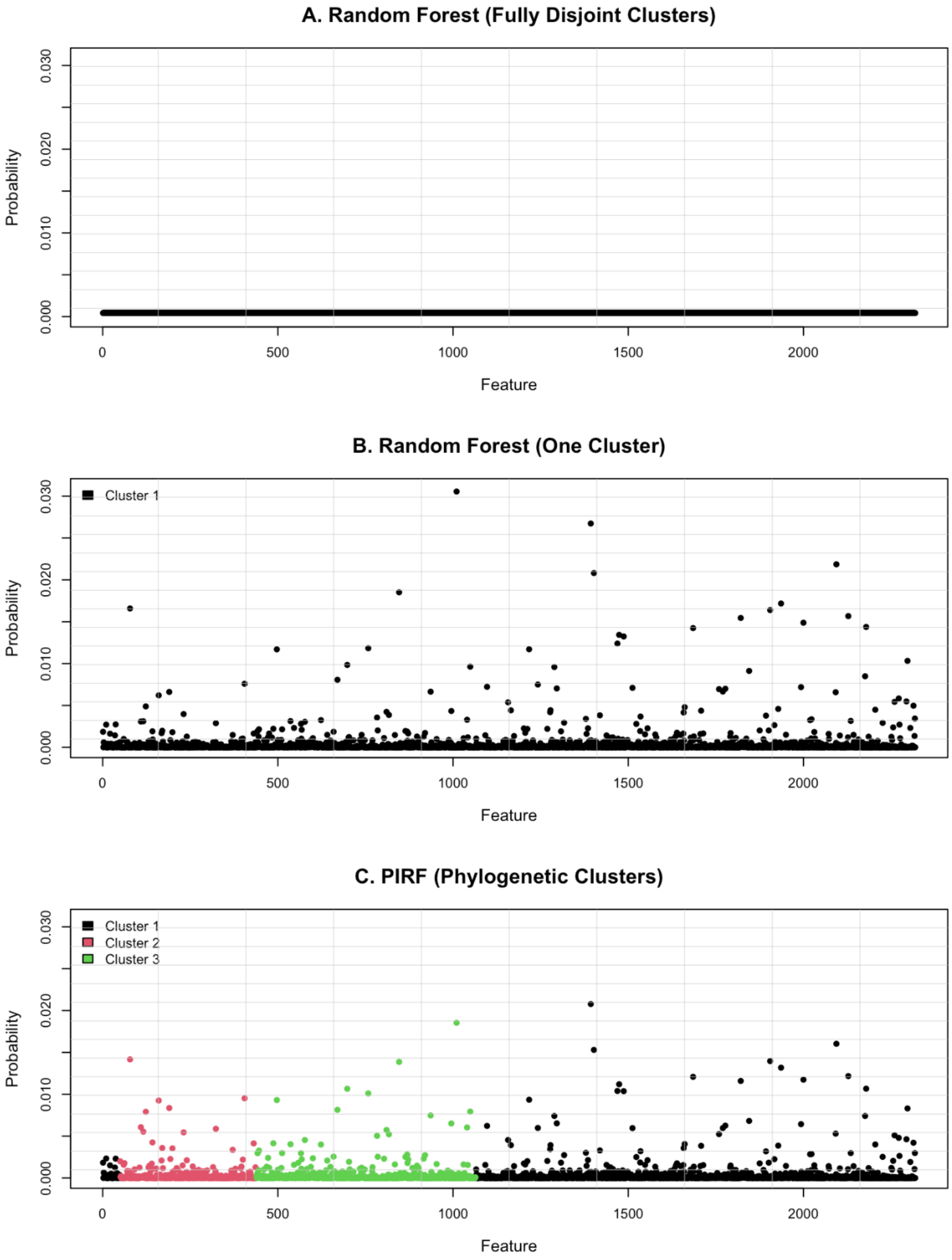

S6 Fig. Visual representation of the selection probabilities for the regression task of Age (Gut) using three methods: A. Random Forest (Fully Disjoint Clusters); B. Random Forest (One Cluster); and C. PIRF (Phylogenetic Clusters). Within each method, the probabilities sum to one; as such, Random Forest (Fully Disjoint Clusters) shows the least variability, Random Forest (One Cluster) shows the highest variability, and PIRF (Phylogenetic Clusters) shows an intermediate level of variability.

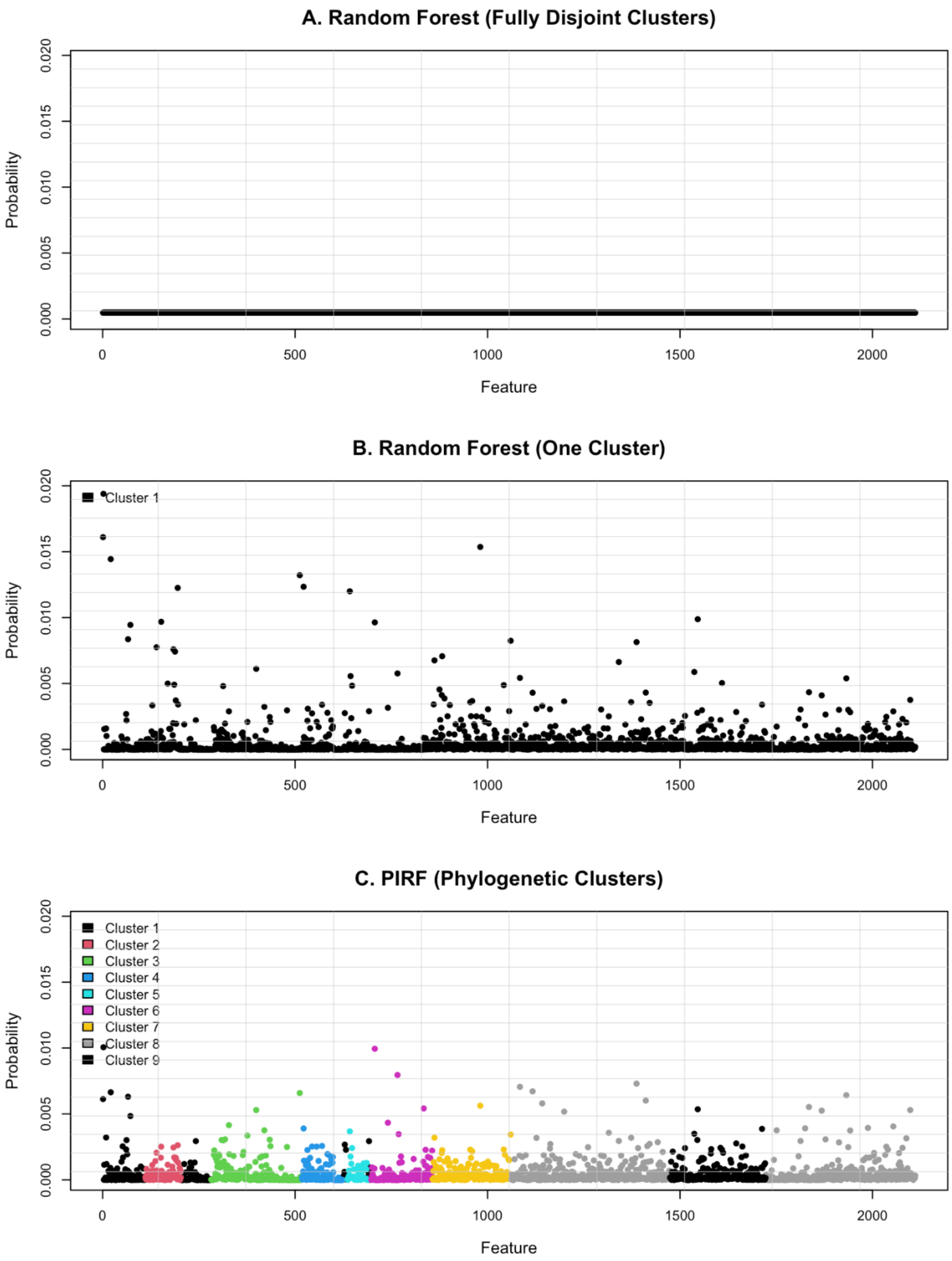

Supplement: Supplemental material — Figures S1 to S6. [file spectrum.03451-25-s0001.pdf]
